# A Key Role for Chd1 in Histone H3 Dynamics at the 3′ Ends of Long Genes in Yeast

**DOI:** 10.1371/journal.pgen.1002811

**Published:** 2012-07-12

**Authors:** Marta Radman-Livaja, Tiffani K. Quan, Lourdes Valenzuela, Jennifer A. Armstrong, Tibor van Welsem, TaeSoo Kim, Laura J. Lee, Stephen Buratowski, Fred van Leeuwen, Oliver J. Rando, Grant A. Hartzog

**Affiliations:** 1 Department of Biochemistry and Molecular Pharmacology, University of Massachusetts Medical School, Worcester, Massachusetts, United States of America; 2 Department of Molecular, Cell, and Developmental Biology, University of California Santa Cruz, Santa Cruz, California, United States of America; 3 W. M. Keck Science Department, Scripps, Claremont McKenna, and Pitzer Colleges, Claremont, California, United States of America; 4 Division of Gene Regulation, Netherlands Cancer Institute and Netherlands Proteomics Centre, Amsterdam, The Netherlands; 5 Department of Biological Chemistry and Molecular Pharmacology, Harvard University, Boston, Massachusetts, United States of America; The University of North Carolina at Chapel Hill, United States of America

## Abstract

Chd proteins are ATP–dependent chromatin remodeling enzymes implicated in biological functions from transcriptional elongation to control of pluripotency. Previous studies of the Chd1 subclass of these proteins have implicated them in diverse roles in gene expression including functions during initiation, elongation, and termination. Furthermore, some evidence has suggested a role for Chd1 in replication-independent histone exchange or assembly. Here, we examine roles of Chd1 in replication-independent dynamics of histone H3 in both *Drosophila* and yeast. We find evidence of a role for Chd1 in H3 dynamics in both organisms. Using genome-wide ChIP-on-chip analysis, we find that Chd1 influences histone turnover at the 5′ and 3′ ends of genes, accelerating H3 replacement at the 5′ ends of genes while protecting the 3′ ends of genes from excessive H3 turnover. Although consistent with a direct role for Chd1 in exchange, these results may indicate that Chd1 stabilizes nucleosomes perturbed by transcription. Curiously, we observe a strong effect of gene length on Chd1's effects on H3 turnover. Finally, we show that Chd1 also affects histone modification patterns over genes, likely as a consequence of its effects on histone replacement. Taken together, our results emphasize a role for Chd1 in histone replacement in both budding yeast and *Drosophila melanogaster*, and surprisingly they show that the major effects of Chd1 on turnover occur at the 3′ ends of genes.

## Author Summary

Nucleosomes prevent transcription by interfering with transcription factor binding at the beginning of genes and blocking elongating RNA polymerase II across the bodies of genes. To overcome this repression, regulatory proteins move, remove, or structurally alter nucleosomes, allowing the transcription machinery access to gene sequences. Over the body of a gene, it is important that nucleosome structure be restored after a polymerase has passed by; failure to do so may lead to activation of transcription from internal gene sequences. Interestingly, although nucleosomes constantly move on and off of promoters, they are relatively stable over the bodies of genes. Thus, the same nucleosomes that are removed to allow a polymerase to pass by must be reassembled in its wake. Here, we examine the role of an ATP–dependent chromatin remodeling protein, Chd1, in regulating nucleosome dynamics. We find that Chd1 is important for exchange of the histone H3 in both yeast and *Drosophila* and that, surprisingly, while it promotes exchange of histones at the beginning of genes, it prevents exchange at the ends of genes. Finally, we show that Chd1 helps determine the characteristic pattern of chemical modifications of histone H3 found over actively transcribed gene sequences.

## Introduction

Eukaryotic genomes are packaged as chromatin, whose fundamental repeating subunit, the nucleosome, is composed of 147 bp of DNA wrapped 1.7 times around an octameric histone core. Nucleosomes may interact with each other to form higher-order levels of chromatin packaging necessary to compact an entire genome within a nucleus. This genome packaging strategy leads to a dominant theme in eukaryotic gene regulation: nucleosomes tend to repress gene expression, and a large array of gene regulatory mechanisms in eukaryotes operate by strengthening or weakening the repressive effects of nucleosomes on gene expression [1].

Genome-wide nucleosome mapping studies indicate that although the majority of a eukaryotic genome is typically covered with regularly spaced nucleosomes, nucleosome depleted or nucleosome free regions are frequently found over promoters and at the 3′ ends of genes (reviewed in [2]). Although these studies give a fixed snapshot of chromatin organization, other analyses indicate that chromatin is dynamic. Studies in which histones were pulse-labeled with radioisotopes or tagged with GFP demonstrated that histones can be actively exchanged on chromatin, even in the absence of DNA replication [3], [4]. More recent work has utilized induction of epitope-tagged alleles of histones in G1-arrested yeast cells followed by chromatin immunoprecipitation to examine histone H3 dynamics genome-wide [5], [6]. These studies show that histone H3 exchanges at a high rate on promoters and in other intergenic regions such as downstream of the 3′ ends of genes. With the exception of highly-transcribed genes, the bodies of genes, even those that are transcribed at moderate rates, exhibit much lower H3 exchange rates.

Although nucleosomes over transcribed genes appear to be relatively stable *in vivo*, nucleosomes form a strong barrier to elongating RNA polymerase II (RNA Pol2) *in vitro* [7]. Thus, it is likely that accessory factors assist in transcription elongation to alleviate this barrier. These factors may promote the temporary disassembly or displacement of nucleosomes permitting the passage of elongating RNA Pol2, and furthermore, they may assist in nucleosome (re)assembly after polymerases have passed. A wide variety of factors have been implicated in the dynamics and maintenance of chromatin structure over transcribed sequences. These include ATP-dependent chromatin remodeling enzymes, enzymes that post-translationally modify histones, histone chaperones and transcription elongation factors [8]. Interestingly, mutations affecting a number of these factors cause a cryptic transcription initiation phenotype, in which disruption of chromatin in the body of genes leads to activation of internal, normally quiescent promoters [9].

One factor implicated in the regulation of transcribed chromatin is the ATP-dependent chromatin remodeling enzyme Chd1. Chd1 is the founding member of a family of highly conserved chromatin remodeling enzymes found throughout eukaryotes [10]. Although budding yeast only express a single Chd1 protein, at least 9 CHD family proteins are expressed in humans. Mammalian CHD family members have been implicated in diverse roles including promotion of normal organismal development, and the maintenance of pluripotency and prevention of heterochromatin formation in mouse embryonic stem cells [10]. In addition, mutations in CHD protein genes are implicated in several human cancers and CHARGE syndrome, which is characterized by a phenotypically heterogeneous set of developmental defects [10], [11].

CHD proteins typically have a pair of N-terminal chromodomains, a central Snf2/Swi2 type helicase domain and a C-terminal domain that mediates DNA or nucleosome binding [10]. The chromodomains of human Chd1 bind histone H3 tails methylated at lysine 4 (H3K4me) suggesting a mechanism for recruitment [12], [13]. However, yeast Chd1 does not bind H3K4-methylated tails [13], and in *Drosophila melanogaster*, the chromodomains do not play an important role in its localization to chromatin [14]. Recent structural and biochemical studies suggest that rather than mediating chromatin localization, the chromodomains may regulate enzyme activity [15]. *In vitro* assays show that Chd1 has the ability to assemble, remodel, slide and promote regular spacing of nucleosomes [16]–[18]. Chromatin immunoprecipitation in budding and fission yeast, and immunostaining of *Drosophila* polytene chromosomes show that Chd1 associates with both promoters and transcribed regions of active genes [19]–[23]. Consistent with its localization on genes, genetic studies in yeast have implicated Chd1 in the regulation of transcription initiation, elongation and termination [22], [24]–[28]. Although Chd1 can be purified as a monomer, its association with several complexes that regulate initiation and elongation, which include mediator, FACT, the Paf1 complex, SAGA and SLIK, provides further support to these conclusions [22], [29]–[33]. Chd1 also associates with histone chaperones Nap1 in fission yeast, and HirA, a histone chaperone for histone H3.3, in fruit flies [19], [34].

Several studies suggest mechanisms for how Chd1's biochemical activity may relate to these biological functions. Chd1 can promote transcription and catalyze activator dependent, promoter specific nucleosome remodeling *in vitro* [35], [36]. Furthermore, in *Schizosaccharomyces pombe*, Chd1 (Hrp1) acts at a subset of promoters to disassemble nucleosomes close to the transcription initiation site [19]. In *Drosophila*, following fertilization of an egg, sperm chromatin is decondensed, protamines are removed and replaced with nucleosomes whose only form of histone H3 is the replication-independent variant H3.3 [37]. Interestingly, in *chd1* mutants, H3.3 levels in decondensing sperm chromatin are greatly reduced and unevenly distributed, suggesting a role for Chd1 in the replication-independent assembly or distribution of H3.3 nucleosomes [34], [38].

A recent high-resolution genome-wide nucleosome mapping study in budding yeast points to an *in vivo* role for Chd1's nucleosome remodeling activity. Nucleosomes are typically regularly positioned over genes in wild type yeast cells [39]. However, in a *chd1*Δ** mutant, this positioning is largely lost over gene bodies [40]. Specifically, nucleosome free regions at the 5′ and 3′ ends of genes and the first (+1) nucleosome over the transcribed region were minimally affected by loss of Chd1, but downstream nucleosomes (particularly those starting at the +3 position) were dramatically delocalized in *chd1*Δ** yeast cells. Curiously, micrococcal nuclease digestion patterns of bulk chromatin are not affected in a *chd1* mutant, suggesting that Chd1 affects the positioning of nucleosome arrays primarily over the transcribed body of genes, rather that the precise spacing between any given pair of nucleosomes [40], [41]. Although *chd1* mutations have modest effects on gene expression in yeast, and are virtually indistinguishable from wild type strains in phenotypic assays, they do cause a cryptic initiation phenotype, consistent with the loss of nucleosome organization over the body of genes [9], [28], [42], [43].

Although these data clearly demonstrate a role for Chd1 in nucleosome positioning *in vivo*, the mechanism underlying its *in vivo* function and its relationship to transcription remains unclear. In this study, we examine the role(s) of Chd1 in governing the replication-independent exchange of newly-expressed histone H3 onto chromatin in budding yeast and Drosophila using genome-wide methodologies. *Chd1* mutants have dramatic defects in the localization of the replication-independent histone variant H3.3 in flies, while in *Saccharomyces cerevisiae*, *chd1*Δ** mutants exhibit dramatic defects in H3 turnover in coding regions. Surprisingly, Chd1 predominantly affects histone H3 exchange at the 3′ ends of coding regions, and this effect on turnover depends on gene length – H3 turnover at 3′ ends is fairly concordant between wild type and *chd1*Δ** strains for genes 1 kb and shorter, whereas Chd1 appears to specifically stabilize nucleosomes over the 3′ ends of longer genes. Finally, we show that loss of Chd1 globally alters histone modification patterns related to active transcription, with H3K36me3 in particular shifting in concert with the changed patterns of H3 replacement. Together, our results show that Chd1 plays a key role in histone H3 dynamics, and surprisingly, that yeast Chd1's influence on H3 dynamics is most apparent at the 3′ ends of genes.

## Results

### Chd1 Influences Replication-Independent Assembly of H3.3 in *Drosophila*

Previously, Fyodorov and colleagues examined the distribution of epitope-tagged, full length H3.3 in the *Drosophila* syncytial blastoderm and only observed a modest defect in H3.3 distribution in *chd1* null mutants [34]. Because the H3.3 N-terminal tail, which is required for replication-dependent assembly of H3.3 [44], was intact in this experiment, we reasoned that any defect in replication-independent assembly of the tagged H3.3 might have been obscured.

**Figure 1:**
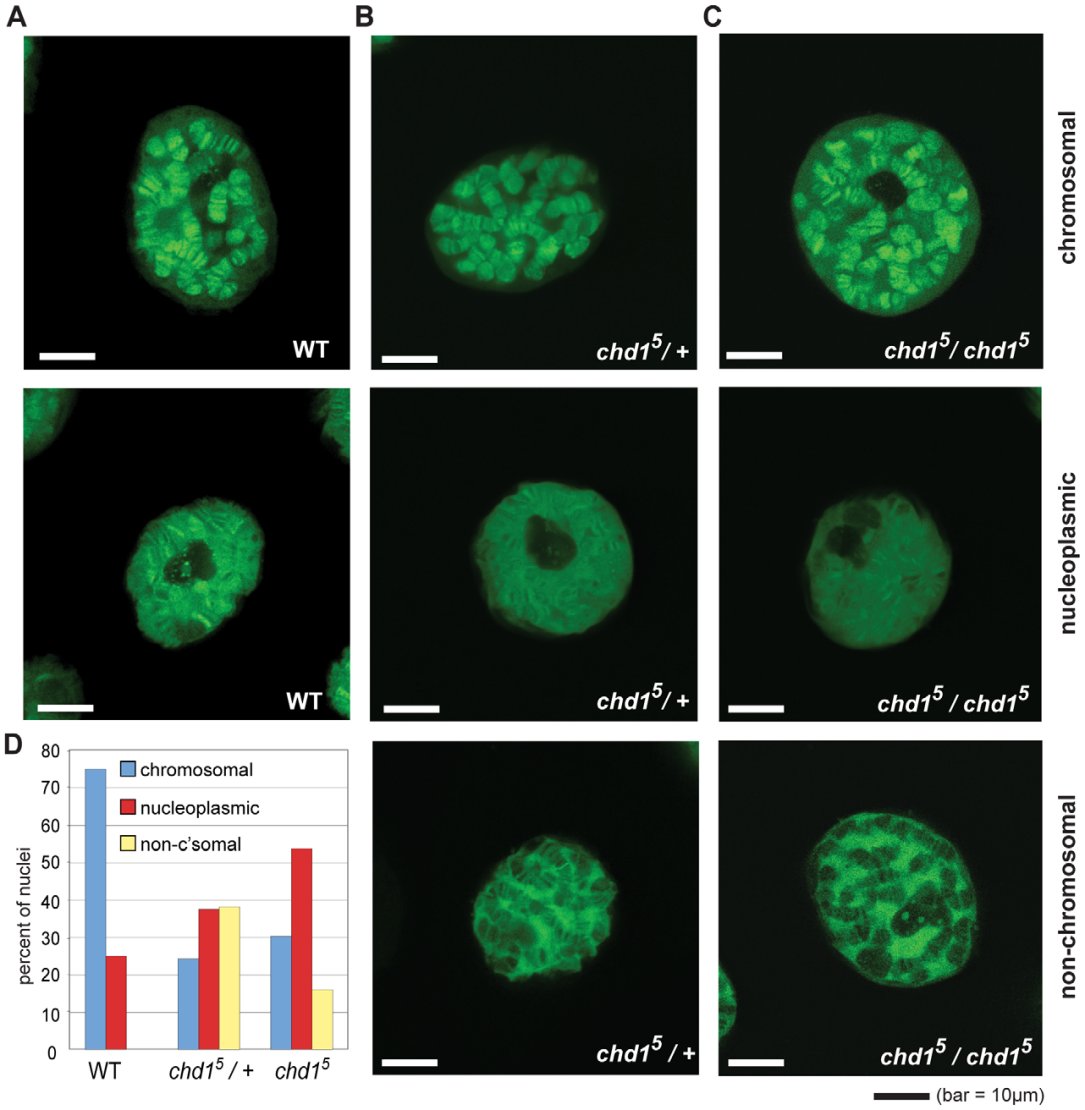
Chd1 affects H3.3_core_-GFP localization on Chd1 in *Drosophila*. (A) Representative sections from confocal imaging of H3.3_core_-GFP in nuclei from salivary glands of wild type larvae, (B) *chd1^5^* heterozygotes and (C) *chd1^5^* homozygotes. The GFP signal is pseudo green. In all cases, H3.3_core_-GFP was expressed from *P[UHS-H3.3_core_-GFP]* and driven by *P{GawB} AB1-Gal4*. (D) Quantitation of banding patterns observed in nuclei from flies with the indicated genotypes. A total of 44 wild type, 144 heterozygote, and 162 homozygous null nuclei were scored, all blind to genotype.

To reassess Chd1's role in replication-independent deposition of H3.3, we imaged GFP-tagged histone H3.3_core_ in live salivary glands from *chd1* mutant larvae. We utilized a transgenic fly expressing an *AB1*-*GAL4* driver and a Gal inducible histone H3.3_core_-GFP [44]. Because the H3.3_core_ protein encoded by the transgene lacks the N-terminal tail, it is only incorporated into chromatin via the replication-independent pathway [44]. In an otherwise wild type background, H3.3_core_-GFP was deposited into the polytene chromosome arms of salivary glands (Figure 1A). In some cases, we also observed a nucleoplasmic GFP signal in which the entire nucleus, including non-chromosomal territories, exhibited a strong GFP signal, although a chromosomal banding pattern was still evident. In flies that were heterozygous or homozygous for *chd1^5^*, a null allele of Chd1 [21], we observed salivary gland nuclei with GFP signals similar to those of wild type, *i.e.* chromosomal or broad nucleoplasmic GFP fluorescence. However, we also observed nuclei with a novel, “non-chromosomal” phenotype where the polytene arms appear almost devoid of GFP signal and a substantial nuclear, non-chromosomal H3.3_core_-GFP signal was still apparent (Figure 1B, 1C). We determined the relative frequencies of these phenotypes in wild type and mutant flies by blind scoring, and observed that the predominant chromosomal fluorescence pattern observed in wild type cells declined dramatically in *chd1* mutants, whereas the nucleoplasmic and non-chromosomal patterns increased in frequency (Figure 1D). We observed similar phenotypes when we repeated these experiments with independently derived *chd1^5^* flies using a different balancer chromosome (data not shown). These results do not appear to be due to any peculiarity of the *AB1-GAL4* driver as we observed similar fluorescence patterns when we used *sgsGAL4* and *eyelessGAL4* drivers (data not shown). Furthermore, we did not observe obvious differences in the strength of H3.3_core_-GFP signals between flies with the three analyzed genotypes (wild type, *+*/*chd1^5^* heterozygous and *chd1^5^*/*chd1^5^* homozygous), nor between nuclei with the three observed staining patterns (chromosomal, non-chromosomal and nucleoplasmic) (Figure S1), suggesting that the observed localization patterns were not due to differences in H3.3_core_-GFP expression. Rather, we favor the idea that the variability observed here reflects perdurance of maternally contributed Chd1, which has been observed previously [21]. Immunostaining of fixed polytene chromosomes similarly revealed a reduction of H3.3_core_-GFP on chromosomes derived from *chd1^5^* mutant larvae, while levels of full length H3.3-GFP were not affected by loss of Chd1 (Figure S2), consistent with the ability of full length H3.3 to incorporate through both replication-dependent and –independent pathways. Consistent with our observations in the *chd1^5^* mutants, we observed decreased association of H3.3_core_-GFP with polytene chromosomes when we knocked down Chd1 levels with either of two RNAi constructs (Figure S2 and data not shown). Overall, these data are consistent with the possibility that Chd1 may contribute to replication-independent assembly of H3.3 containing nucleosomes.

### The H3 Tail and Chd1 Function Redundantly in Yeast

**Figure 2:**
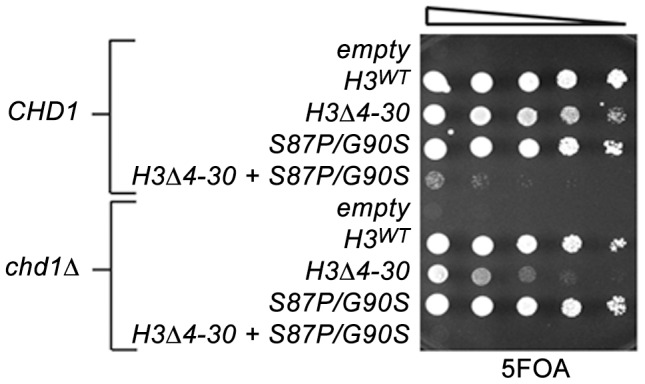
The H3 N-terminal tail functions redundantly with Chd1 and an H3.3-like surface of histone H3 in budding yeast. The indicated histone H3 plasmids (which also carried histone H4) were transformed into wild type *CHD1* or *chd1* null strains that lack both chromosomal copies of the histone H3/H4 genes and contained a *URA3 H3/H4* plasmid. Cultures were adjusted to 1×10^7^ cells per ml and five-fold serial dilutions were spotted directly onto 5FOA media, selecting for cells that had lost the *URA3 H3/H4* plasmid, and incubated for 2 days at 30°C.

To further examine roles of Chd1 in nucleosome dynamics *in vivo*, we turned to budding yeast. To test the idea that Chd1 may modulate replication-independent nucleosome assembly or dynamics, we took advantage of the observation that the yeast H3 N-terminal tail is important for normal chromatin structure [45]. Reasoning that the H3 N-terminal tail deletion mutation likely interferes with replication-dependent assembly of H3, as is the case in *Drosophila* and *Physarum polycephalum*, [44], [46], we predicted that loss of this function would sensitize cells to defects in other chromatin assembly or maintenance pathways, we used a plasmid shuffle strategy to create *CHD1^+^* and *chd1*Δ** yeast strains expressing either wild type histone H3 (H3^WT^) or a histone H3 N-terminal deletion mutation, *H3*Δ*4-30*. Consistent with prior observations, the *chd1*Δ* H3^WT^* strain grew indistinguishably from wild type cells, and the *CHD1 H3*Δ*4-30* strain exhibited a moderate growth defect (Figure 2). Interestingly, the *chd1*Δ* H3*Δ*4-30* double mutant grew much more poorly than the *CHD1 H3*Δ*4-30* single mutant, indicating that Chd1 and the N-terminal tail of H3 share a redundant function.

In contrast to other model organisms, the budding yeast genome expresses only a single non-centromeric form of histone H3. However, the major histone H3/H4 chaperones, including the H3.3 chaperone HirA, are conserved, suggesting that yeast retain distinctive replication dependent and independent chromatin assembly pathways [47]. We have obtained data consistent with this idea in a screen for genetic suppressors of a cold-sensitive allele of transcription elongation factor *SPT5*. Among these suppressors were mutations in *CHD1*, mutations in the H3K4 and H3K36 histone methyltransferases *SET1* and *SET2*, histone H3K4 and H3K36 substitutions, and mutations in members of the RPD3S histone deacetylase complex. Further characterization of these suppressors led us to propose that they act by lowering the chromatin barrier to efficient transcription elongation [28].

Given the observations described above, we recently screened a randomly mutagenized plasmid library for histone H3 mutations that suppress *spt5Cs*- (to be described in detail elsewhere). Among the suppressor mutations obtained in that screen, we isolated a mutation, H3-S87P/G90S, which simultaneously alters two of the four residues that distinguish histone H3.1 from H3.3 in other eukaryotes. Yeast expressing the S87P/G90S form of histone H3 from the normal *HHT2* locus are viable, indicating that this mutation is unlikely to strongly perturb replication coupled chromatin assembly. As with several other of the mutations that suppress *spt5Cs*- (*e.g.*, H3K36R, *set2*, mutations affecting Rpd3s), the H3-S87P/G90S mutant caused cryptic initiation of transcription (Figure S3).

We therefore examined genetic interactions between the *H3-S87P/G90P*, *chd1*Δ** and the *H3*Δ*4-30* mutations using the plasmid shuffle assay described above (Figure 2). Interestingly, the *chd1*Δ* H3-S87P/G90S* double mutant exhibited no new mutant phenotypes, whereas combining *H3-S87P/G90S* with the *H3*Δ*4-30* deletion resulted in a very poor growth phenotype and the *chd1*Δ* H3-S87P/G90S H3*Δ*4-30* triple mutation showed an even more severe growth defect. Thus, like Chd1, residues 87 and 90 of histone H3 function redundantly with the H3 N-terminal tail. It is tempting to argue that these data indicate that Chd1 interacts with histone H3 via a surface defined by residues 87 and 90. However, the fact that the phenotype of the *chd1*Δ* H3-S87P/G90S H3*Δ*4-30* triple mutant is more severe than that of the *chd1*Δ* H3*Δ*4-30* double mutant suggests that H3 residues S87 and G90 may retain functions that are redundant with the H3 tail, even when Chd1 is absent.

### Chd1 Affects Histone H3 Dynamics

The data presented above suggest that Chd1 affects replication independent dynamics of histone H3. To test this idea directly in budding yeast, we used a yeast strain carrying galactose-inducible Flag-tagged H3, coupled with chromatin immunoprecipitation and tiling microarray (ChIP on chip) analysis, to follow the incorporation of newly-synthesized H3 genome-wide in cells arrested in the cell cycle [6]. Briefly, wild type or *chd1*Δ** yeast strains are arrested in G1 phase using alpha factor, then Flag-H3 is induced with galactose, and after 60 minutes Flag-H3 and total H3-associated DNA are subject to ChIP enrichment and competitively hybridized on ∼250 bp resolution tiling microarrays. Resulting Flag/total H3 ratios provide locus-specific estimates of H3 turnover rates.

**Figure 3:**
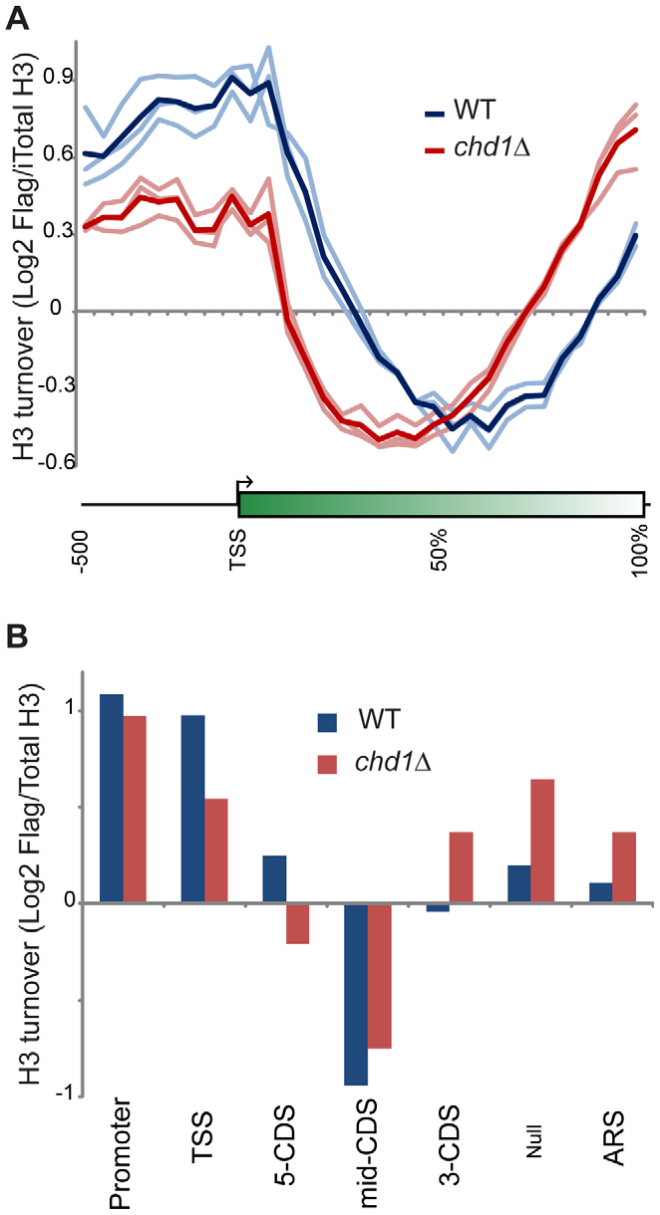
Chd1 plays a key role in H3 replacement dynamics at gene ends. (A) H3 replacement was measured in G1-arrested cells by induction of Flag-H3 for 60 minutes, followed by ChIP enrichment of both Flag-containing and total H3-associated DNA and subsequent competitive hybridization to tiling microarrays. H3 turnover is represented as log2 of Flag-H3 ChIP enrichment over total-H3 ChIP (y axis). Here, data for all yeast genes is shown in a “metagene” view, with 10 bins of 50 bp each, upstream of the +1 nucleosome, followed by 20 bins representing 5% increments along gene coding regions, scaling for gene length. Three independent replicate experiments are shown along with the averaged profile, as indicated. (B) Average Flag-H3/total-H3 for various classes of genomic element, defined as in [6], [54]. Briefly, 5′ and 3′ CDS refer to probes within the first and last 500 bp of coding regions, with mid-CDS encompassing any remaining probes. TSS indicated probes up to 500 bp upstream of the ATG, and Promoter includes all remaining upstream probes. ARS includes all probes within 200 bp of an ARS. Null indicates all remaining probes, predominantly those that fall between convergently-transcribed genes.

Figure 3A shows a “metagene” analysis of H3 turnover in 3 biological replicate samples for wild type (blue) and *chd1*Δ** (red) strains. The wild type profile recapitulates previous results from multiple labs [5], [6], [48] – H3 replacement is highest over promoters and at the 5′ ends of genes, with coding regions being remarkably protected from H3 replacement, and modest levels of turnover being seen at the 3′ ends of genes.

Conversely, *chd1*Δ** mutants exhibit H3 turnover patterns in which genes appear to effectively reverse polarity. Turnover is still lowest over coding regions, but the trough of minimal turnover has shifted 5′ along coding regions. Promoter and 5′ turnover are slower in *chd1*Δ** cells, whereas maximal H3 replacement is instead observed at the 3′ ends of genes. This behavior is highly unusual, as several published [5], [6], [49], [50] and a large number of unpublished (OJR, unpublished data) mutants exhibit quite distinct turnover defects. We confirmed the increased 3′ H3 replacement at two model genes (Figure S4) using an entirely independent assay for histone replacement based on Cre-mediated recombination of C-terminal H3 epitope tags [51]–[53].

As a separate visualization, Figure 3B shows the average H3 turnover for various classes of genomic elements [6], [54]. Even though a previous microarray analysis showed only a very modest effect of *chd1*Δ** on transcription [18], we considered the possibility that the altered H3 turnover in *chd1* cells could be due to a large shift in cellular transcription. However, we observed strong concordance of ChIP on chip of RNA Pol2 signals for wild type and *chd1*Δ** cells (Figure S5). Moreover, as noted below, Chd1's effects on H3 replacement are strongly gene length-dependent, but we find no correlation between mRNA abundance changes and gene length or transcription frequency (Figure S6). Thus, Chd1's effects on turnover are not secondary effects of altered transcription.

### Chd1 Protects Long Genes from 3′ H3 Replacement

We sought to understand what factors might contribute to Chd1 recruitment or function at gene ends. To this end, we first examined the genes with the greatest changes in H3 replacement at their 3′ ends in *chd1*Δ** mutants. Notably, we observed that the genes with the greatest changes in 3′ end H3 turnover were among the longest (>3 kb) genes in budding yeast. We therefore systematically analyzed the effects of gene length on Chd1's role in H3 replacement.

**Figure 4:**
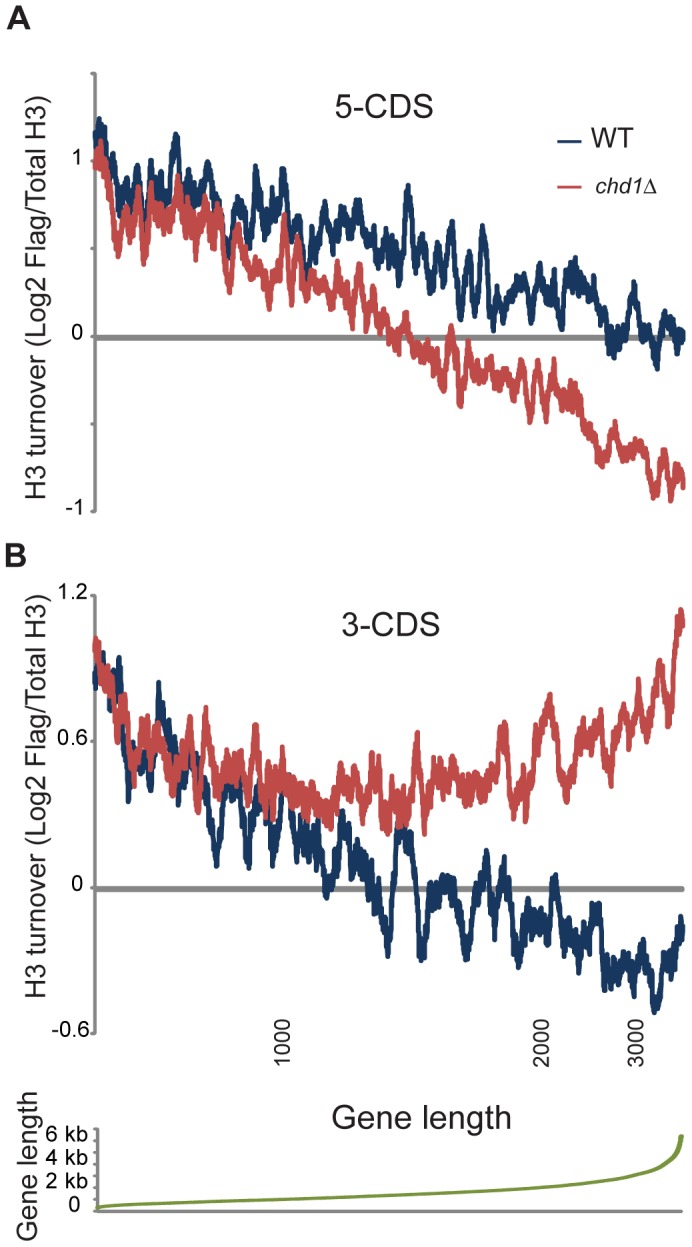
Length dependence of Chd1 effects on H3 replacement. H3 replacement was averaged for the 500 bp at the 5′ ends of genes (A), or the 3′ ends of genes (B). Genes were ordered by length, and an 80 gene window average is shown for wild type and *chd1Δ* turnover data as indicated. Bottom panel plots gene lengths, and locations for 1, 2, and 3 kb are indicated below panel (B).

Figure 4 shows H3 turnover levels for wild type and *chd1*Δ** yeast cells at gene ends (the first and last 500 bp of coding regions) as a function of gene length. At both gene ends there is strong length dependence for H3 turnover in wild type yeast cells, with turnover decreasing as a function of gene length. Notably, for both 5′ end and 3′ end H3 turnover, Chd1's effect on H3 turnover was greatest at unusually long genes. In addition, we found that Chd1's effect on 3′ turnover was greater at highly transcribed genes (Figure S7).

The length dependence for 3′ end H3 replacement (Figure 4B) is particularly remarkable – H3 turnover is nearly identical in wild type and *chd1*Δ** strains for genes of up to roughly 1 kb in length, at which point 3′ end turnover continues to decrease with gene length in wild type cells but stays essentially constant in *chd1*Δ** cells. In other words, the role of Chd1 in wild type cells seems to be to help stabilize nucleosomes at the 3′ ends of genes over 1 kb in length.

### Chd1 Effects on Histone Modification Patterns

Chd1's effects on H3 turnover are greatest at genomic loci that are enriched in H3K36me3 or H3K4me3 modified nucleosomes [55], and *chd1* mutants exhibit synthetic genetic interactions with the H3K4 and H3K36 methyltransferases Set1 and Set2 [56], [57]. We therefore determined if *chd1*Δ** mutants affect histone modification patterns by genome-wide mapping of H3K4me3 and H3K36me3 in wild type and *chd1*Δ** yeast cells. Crosslinked chromatin from these two strains was digested with micrococcal nuclease, immunoprecipitated with H3K4me3 or H3K36me3 antisera and competitively hybridized to microarrays with micrococcal nuclease digested input DNA.

**Figure 5:**
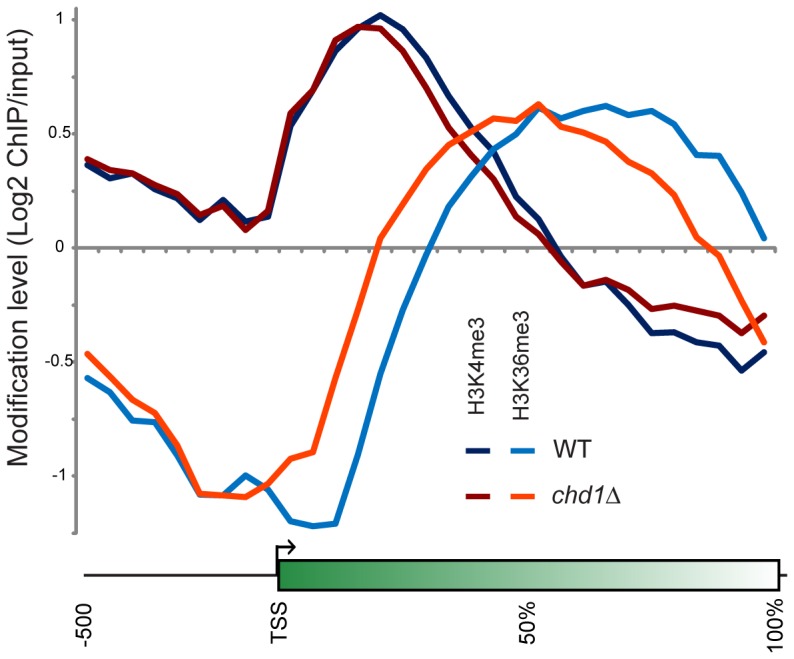
Chd1 effects on H3 methylation patterns. H3K4me3 and H3K36me3 were mapped genome-wide by ChIP-chip on tiling microarrays. Metagene analysis is shown for wild type and *chd1Δ* strains, as indicated.

Figure 5 shows average H3K4me3 and H3K36me3 patterns in *chd1*Δ** cells. On average, H3K4me3 patterns were minimally affected by loss of Chd1, although we noticed a subtle increase in H3K4me3 at the 3′ ends of many genes. This may be a consequence of the fact that *chd1*Δ** mutants show increased transcription from “cryptic” internal promoters [9],[28]. Interestingly, the gain in H3K4me3 at the 3′ ends of genes was greatest at longer genes (Figure S8), which also exhibited the greatest defects in H3 turnover.

More dramatically, H3K36me3 patterns were extensively altered in *chd1*Δ** cells, with loss of H3K36me3 at the 3′ ends of genes and a shift in the H3K36me3 peak towards the 5′ ends of genes. Consistent with the loss of H3K36me3 at the 3′ ends of genes, we previously observed increased H3K9/K14 acetylation at the 3′ ends of several genes in a *chd1*Δ** mutant [28], as would be expected since reduced H3K36me3 results in reduced recruitment or activity of the Rpd3S deacetylase complex [27], [58], [59].

In our prior study, we did not observe any significant change in total levels of H3K4me3 or H3K36me3 in a *chd1* mutant [28]. As H3K36me3 typically anticorrelates with H3 turnover [6], we hypothesize that the altered H3K36me3 profile observed here is a consequence of Chd1's effects on H3 turnover – increased H3 turnover at 3′ ends of genes likely results in loss of H3K36me3 at these regions. Consistent with this hypothesis, we found that loss of 3′ H3K36me3 was greatest at longer genes (Figure S8).

## Discussion

We present evidence that Chd1 modulates replication-independent turnover of histone H3 in both *Drosophila* and budding yeast. Chd1's effects on H3 turnover are greatest at genomic loci that normally coincide with peaks of H3K4me3 and H3K36me3 modified nucleosomes. This observation is consistent with prior reports that Chd1 reduces nucleosome density at promoters, can catalyze activator-dependent nucleosome removal and promote transcription *in vitro*, and that it modulates the efficiency of transcription termination [19], [24], [36].

Chd1's effects on H3 turnover may reflect a direct role in histone eviction or deposition during replication-independent histone exchange, consistent with its ability to catalyze ATP-dependent assembly of nucleosomes *in vitro*, or it could reflect a role for Chd1 in stabilization of pre-existing nucleosomes. Here, we observed that the predominant effect of Chd1 on H3 turnover in budding yeast was to repress turnover over the 3′ ends of genes. While we do not yet understand the mechanism underlying this observation, we favor the idea that Chd1 acts upon nucleosomes that have been perturbed by elongating RNA polymerase II, restoring them to their normal structures or positions and thereby stabilizing them. Importantly, we do not favor the alternative model, that Chd1's effects on chromatin are secondary to perturbation of transcription; we and others do not observe significant alterations of gene expression in *chd1* mutants in yeast (Figure S6 and [21]) and Pol II phospho-Ser2 staining of *Drosophila* polytene chromosomes is normal in *chd1* mutants [21].

Chd1's effects on H3 turnover at the 3′ end of genes depended strongly upon gene length (Figure 4), and was also correlated with transcription rate (Figure S7). Given the model above, it is possible that in the absence of Chd1, perturbation of nucleosome positioning by transcription complexes increases with gene length and nucleosome number. Alternatively, Chd1's function may relate to supercoiling changes driven by transcription. To test this we have preliminarily investigated whether additional deletion of the major topoisomerase Top1 affects the chromatin changes observed in *chd1*Δ** yeast mutants. However, we have not observed any suppression of the *chd1*Δ** turnover phenotype in *chd1*Δ*top1*Δ** double mutants (not shown). Thus, at present we have no additional evidence that supercoiling *per se* mediates the length dependence of Chd1 on H3 turnover, although given the ability of other topoisomerases to compensate for loss of Top1 we still consider this an appealing hypothesis.

Previous results show that Chd1 has dramatic effects on nucleosome positioning over coding regions [40]. Our results extend this characterization by showing that Chd1 also has dramatic effects on H3 turnover over coding regions, raising the question of whether these two roles for Chd1 in chromatin structure are related. In other words, does Chd1's effect on H3 replacement follow from its role in establishing wild type nucleosome positions, or vice versa? We have no evidence for either possibility, but note that our prior genetic analyses suggest that *chd1*Δ** mutations lower the nucleosomal barrier to RNA Pol2 elongation [22], [28]. Thus, we speculate that disorganized nucleosomes in *chd1*Δ** mutants could be unusually susceptible to eviction by RNA Pol2. This model is consistent with a recent suggestion that elongating polymerases could cause collisions and eviction of adjacent nucleosomes if they are spaced inappropriately [60]. However, arguing against this are observations that nucleosome ladders are little affected in *chd1* mutants [40], [41]. Future studies will be needed to address these mechanistic questions.

Taken together, our results identify an evolutionarily conserved role for Chd1 in histone turnover in yeast and flies. Most surprising is our finding that the major site of Chd1 function appears to be at the 3′ ends of genes, suggesting that this enzyme may be recruited or regulated by 3′ histone marks such as H3K36me3. Finally, we find that Chd1 largely affects H3 turnover over longer coding regions, raising the question of whether resolving superhelical tension could be a key role for Chd1 in maintaining wild type chromatin architecture.

## Methods

### *Drosophila* Stocks and Crosses

Flies were raised on cornmeal, agar, yeast, and molasses medium, supplemented with methyl paraben and propionic acid. To drive the *P[UHS-H3.3_core_-GFP]* transgene [44], [61] in the salivary gland, flies were crossed to *P{GawB} AB1-Gal4* flies (Bloomington Stock Center). Mutant *chd1^5^* flies were described previously [21]. All crosses were carried out at 18°C.

### Confocal Microscopy

Live analysis of polytene chromosome phenotypes was performed as described previously [62]. To analyze the effect of *chd1^5^* on H3.3*_core_*-GFP incorporation, *chd1^5^ b c sp/BcGla; P[UHS-H3.3_core_-GFP]/TM6B Tb Hu* flies were crossed to *chd1^5^ b c sp/BcGla; P{GawB}AB1/TM6B Tb Hu* flies at 18°C. Flies with *chd1^5^* balanced by *CyO Kr-GFP* instead of *BcGla* were also analyzed and yielded similar results. Salivary glands were dissected and imaged from heterozygous and homozygous *chd1^5^* third instar larvae. For control nuclei, *P{GawB} AB1-Gal4* flies were crossed to *P[UHS-H3.3_core_-GFP]/TM6B Tb Hu* flies. H3.3*_core_*-GFP expression was quantitated by calculating sum pixel intensity in polytene nuclei using the Volocity software package as described previously [62].

### Polytene Chromosome Analysis

Polytene chromosomes were prepared and fixed as described [63] and immunostained using primary antibodies directed against CHD1 ([21], 1∶300 dilution), H5 anti-RNA polymerase II (specific for the Ser 2-phosphorylated form of Pol II CTD, Covance; 1∶50 dilution), and the JL-8 anti-GFP (Clontech, 1∶300 dilution). Secondary antibodies donkey anti-rabbit IgG-Cy3, donkey anti-mouse IgM-Cy2, and donkey anti-mouse IgG Fc2a-DyLight 649 (Jackson ImmunoResearch Laboratories, 1∶200 dilutions) were tested with each individual primary antibody to ensure specificity. Images were examined on an Olympus 1X81 inverted fluorescence microscope and acquired using Image-Pro6.3. Control and mutant chromosomes were photographed using identical exposure times, and images were processed identically in Adobe Photoshop CS3.

### Yeast Strains and Media

All *S. cerevisiae* strains used in this study (see Table S1) were constructed by standard procedures, are isogenic to S288c and are *GAL2+* [64]. Yeast media was made as described previously [65].

### Plasmids

Plasmids used in this study are described in Table S2. Plasmid pJH18-A06 was obtained by random PCR mutagenesis (GAH, TKQ and Araceli Ortiz unpublished). pJH18-**Δ**4-30, S87P/G90S was created by site-directed mutagenesis of pJH18-A06. PGAL-H4-FlagH3 contains a *Kpn*I-*Not*I fragment carrying pGAL-driven Flag-H3 from plasmid MDB61 [50], in pRS416.

### Flag-H3 Expression and Chromatin Immunoprecipitation

Strains transformed with pGAL-H4-FlagH3 were grown to ∼1.2×10^7^ cells/ml in SC-Ura media with raffinose as the carbon source. Cells were G1 arrested with alpha factor and Flag-H3/H4^WT^ expression was induced by addition of galactose (2% final concentration). ChIP assays were preformed as described previously [66]. 60 minutes after addition of galactose, cells were crosslinked with 1% formaldehyde for 15 min, disrupted by bead beating and chromatin was sonicated using a Diagenode Bioruptor to obtain an average size of 500 bp. Chromatin was immunoprecipitated using 40 µl (1∶2 slurry) Anti-Flag M2 Affinity gel (A2220; Sigma) or 1 µg of a rabbit polyclonal antibody against the C-terminus of H3 (ab1791; Abcam). Chelex 100 resin (BioRad) was added to the immunoprecipitated material and Input-DNA samples, and the suspensions were placed at 100°C for 10 min to reverse crosslinks. Samples were treated with proteinase K and DNA was recovered.

Initial characterization and confirmatory analyses of ChIP samples were performed by qPCR in a Corbett Life Science Rotor Gene 6000 machine using SYBR Green as the detection dye (qPCR MasterMix Plus for SYBR Green, Eurogentec). The fold difference between immunoprecipitated material (IP) and total Input sample for each qPCR amplified region was calculated as described in [67], following the formula IP/Input = (2^InputCt - IPCt^). H3 turnover rates were measured as the final ratio between Flag-tagged H3 and total H3 (Flag-H3/Input vs total H3/Input). The sequences of oligonucleotides used in these PCR reactions are listed in Table S3.

The immunoprecipitated DNA was initially PCR amplified using random hexamer primers as described in [68]. The number of cycles used to amplify the samples was adjusted to between 28 and 37 so that there was equal amplification of DNA in the IP vs. Flag-tagged H3 and the IP vs. total H3 samples. Amplified DNA was visualized on a 1% agarose gel and checked for a visible smear of DNA between 500 and 1.2 kB. Amplified DNA from Flag-tagged H3 and total H3 ChIPs samples were labeled and competitively hybridized to tiling microarrays as described below.

### Micrococcal Nuclease Digestion and Chromatin Immunoprecipitation of H3K4me3 and H3K36me3 Chromatin

Wt and *chd1Δ* cells were grown to log phase and fixed with 1% formaldehyde. Cell pellets (from 100 mL cells) were resuspended in 8.8 ml Buffer Z (1 M sorbitol, 50 mM Tris-Cl pH 7.4), with addition 6.5 µl of ß-ME (14.3 M, final conc. 10 mM) and 350 µL of zymolyase solution (10 mg/ml in Buffer Z; Seikagaku America), and the cells were incubated at 30°C shaking at 220 rpm. After spinning at 4000× g, 10 min, 4°C, spheroplast pellets were resuspended in 600 µl NP-S buffer (0.5 mM spermidine, 1 mM ß-ME, 0.075% NP-40, 50 mM NaCl, 10 mM Tris pH 7.4, 5 mM MgCl_2_, 1 mM CaCl_2_) per 100 ml cell culture equivalent. 25–40 units (depending on yeast strain and cell density) of micrococcal nuclease (Worthington Biochemical) were added and spheroplasts were incubated at 37°C for 20 minutes. The digestion was halted by shifting the reactions to 4°C and adding 0.5 M EDTA to a final concentration of 10 mM.

All steps were done at 4°C unless otherwise indicated. For each aliquot, Buffer L (50 mM Hepes-KOH pH 7.5, 140 mM NaCl, 1 mM EDTA, 1% Triton X-100, 0.1% sodium deoxycholate) components were added from concentrated stocks (10–20×) for a total volume of 0.8 ml per aliquot. Each aliquot was rotated for 1 hour with 100 µl 50% Sepharose Protein A Fast-Flow bead slurry (Sigma) previously equilibrated in Buffer L. The beads were pelleted at 3000× g for 30 sec, and approximately 100 µl of the supernatant was set aside for the input sample. With the remainder, antibodies were added to each aliquot (equivalent to 100 ml of cell culture) in the following volumes: 10 µl anti-H3K36me3 (Abcam polyclonal), or 7 µl anti-H3K4me3 (Millipore monoclonal). Immunoprecipitation, washing, protein degradation, and DNA isolation were performed as previously described [69]. The samples were amplified, with a starting amount of up to 75 ng for ChIP samples, using the DNA linear amplification method described previously [54].

### Microarray Hybridization of ChIP'ed Material

2.5 µg of aRNA produced from the linear amplification were labeled via the amino-allyl method as described on www.microarrays.org. Labeled probes (a mixture of Cy5 labeled input and Cy3 labeled ChIP'ed material) were hybridized onto an Agilent yeast 4×44 whole genome array. The arrays were scanned at 5 micron resolution with the Agilent array scanner. Image analysis and data normalization were performed as previously described [54].

### Microarray Data Availability

Microarray data have been deposited in GEO (Accession #GSE38540).

## Supporting Information

Figure S1 The *chd1^5^* mutation does not effect H3.3_core_-GFP expression. Total fluorescent intensity and volume of polytene nuclei were determined by analysis of confocal z-stacks using Volocity software. Average fluorescent intensity/nucleus (arbitrary units; +/− Std. Dev.) are shown for (A), the indicated genotypes and (B), the indicated phenotypes. In (A), *non-Bc* and *non-Kr* refer to the balancer chromosomes in the parental *chd1* strains used to generate the homozygous nulls.
(TIF)

Figure S2 Loss of Chd1 affects localization of H3.3core-GFP but not H3.3-GFP. (A) Levels of H3.3_core_GFP are reduced on polytene chromosomes of *RNAi-chd1 expressing* larvae (*VDRC26277* driven by *AB1-gal4;* [*70*] *)* as compared to those from control larvae. Chd1 (red), Pol IIo^ser2^ (green), GFP (blue), DAPI (white in left panel, not included in merge). (B) Levels of H3.3^core^GFP are reduced on polytene chromosomes derived from *chd1* mutant larvae as compared to control larvae. (C) In contrast, levels of full length H3.3-GFP remain similar on polytenes derived from *chd1* mutant larvae as compared to control larvae. Both *chd1^5^* and *chd1^4^* are null alleles [21]. Chd1 (red), GFP (green), DAPI (white in left panel and blue in merge).
(TIF)

Figure S3 The histone H3-S87P/G90S mutation causes a cryptic initiation phenotype. (A) Diagram of the p*GAL1-FLO8-HIS3* reporter gene. Transcription initiation from the normal *FLO8* start site produces a transcript in which *HIS3* in out of frame and not translated. Internal initiation from within *FLO8* produces in frame transcripts and a His+ phenotype as indicated by growth on media lacking histidine. Strain GHY2010 was transformed with *CEN LEU2 hht2 HHF2* plasmids carrying the indicated mutations and plated to 5FOA to select for cells that had lost the wild type *CEN URA3 HHT2 HHF1* plasmid. These cells were subsequently grown in liquid culture, adjusted to 1×10^7^ cells/ml and 5-fold serial dilutions were spotted to SC-His+Gal media and incubated at 30°C for 3 days. (B) The H3-S87P/G90S mutation causes a cryptic initiation phenotype. (C) Positive and negative controls. As demonstrated previously [28], the H3**Δ**4-30 and H3K36R mutations cause a cryptic initiation phenotype whereas H3K4R mutation does not.
(TIF)

Figure S4 Chd1 affects H3 turnover in an independent turnover assay. (A) Schematic showing the locations of 5′ and 3′ q-PCR primers within the indicated genes. (B) H3 turnover was measured using the system described in [51]. Briefly, yeast carrying H3-HA were arrested by nutrient depletion, and Cre-Lox recombination was used to recombine out the HA tag to yield H3-T7 expression. Yeast were released into cell cycle arrest in benomyl-nocodazole, and H3-HA and H3-T7 were isolated by ChIP. T7/HA ratio (indicating H3 replacement) was calculated by q-PCR at the indicated locations for wild type and *chd1*Δ** yeast, as indicated. As found using the pGAL-based turnover system, loss of Chd1 resulted in increased 3′ turnover over these long coding regions.
(TIF)

Figure S5 Chd1 has minimal effects on global transcription. (A) RNA Pol2 was mapped genome-wide in wild type and *chd1*Δ**, and RNA Pol2 enrichment was averaged for all genes. Scatterplot shows average RNA Pol2 enrichment for all genes, comparing wild type (x axis) and *chd1*Δ** (y axis). (B) 5′ bias in RNA Pol2 (defined as the 5′ RNA Pol2 enrichment over the 3′ RNA Pol2 enrichment) is scatterplotted for wild type and mutant as indicated. Note strong correlation with slope = 1, indicating no systematic bias in RNA Pol2 localization patterns in *chd1*Δ** mutants.
(TIF)

Figure S6 Lack of correlation between transcript length, transcription frequency and changes in mRNA expression in chd1 mutant. (A) Scatterplot comparing a 20 gene moving average of Log2 (mut/WT) *chd1* expression microarray data to transcript length. (B) Scatterplot comparing a 20 gene moving average of Log2 (mut/WT) *chd1* expression microarray data to gene transcription frequency in wild type cells.
(TIF)

Figure S7 Chd1 effects on 3′ histone replacement are greater at highly transcribed genes. The change in histone replacement at the 3′-CDS (last 500 bp of coding regions) between wild type and *chd1*Δ** yeast was calculated, and this value is scatterplotted against gene length. Within this scatterplot, we generated 80 gene running window averages for those genes transcribed at low (green), middle (blue), and high (red) levels in wild type cells based on genome-wide Pol2 ChIP-chip [71]. Note that the red line is consistently higher than the blue or green lines, indicating that after correcting for gene length, genes with higher transcription rates exhibit greater Chd1-dependent stabilization of 3′ nucleosomes.
(TIF)

Figure S8 Histone modification changes correlate with gene length. (A) H3K36me3 levels at the 3′ 1 kb of genes is shown for wild type and mutants, as indicated. Lines show a 50 gene running window average. (B–D) Averaged H3K4me3 and H3K36me3 data for wild type and mutant yeast is shown for short (B), long (C), and extremely long (D) genes. (E) Average gain in H3K36me3 levels at the 3′ 500 bp of genes was averaged for the three indicated gene length classes.
(TIF)

Table S1 Yeast strains used in the study.
(DOC)

Table S2 Plasmids used in this study.
(DOC)

Table S3 Oligonucleotides used for qPCR Analysis of Chromatin Immunoprecipitates. The indicated oligonucleotide pairs were used in initial analyses and verification of histone turnover ChIP experiments.
(DOC)
