# Supplementary figures and images for: A Key Role for Chd1 in Histone H3 Dynamics at the 3′ Ends of Long Genes in Yeast
Source: PLoS Genet. 2012 Jul 12;8(7):e1002811. doi: 10.1371/journal.pgen.1002811 (PMC3395613; doi:10.1371/journal.pgen.1002811)

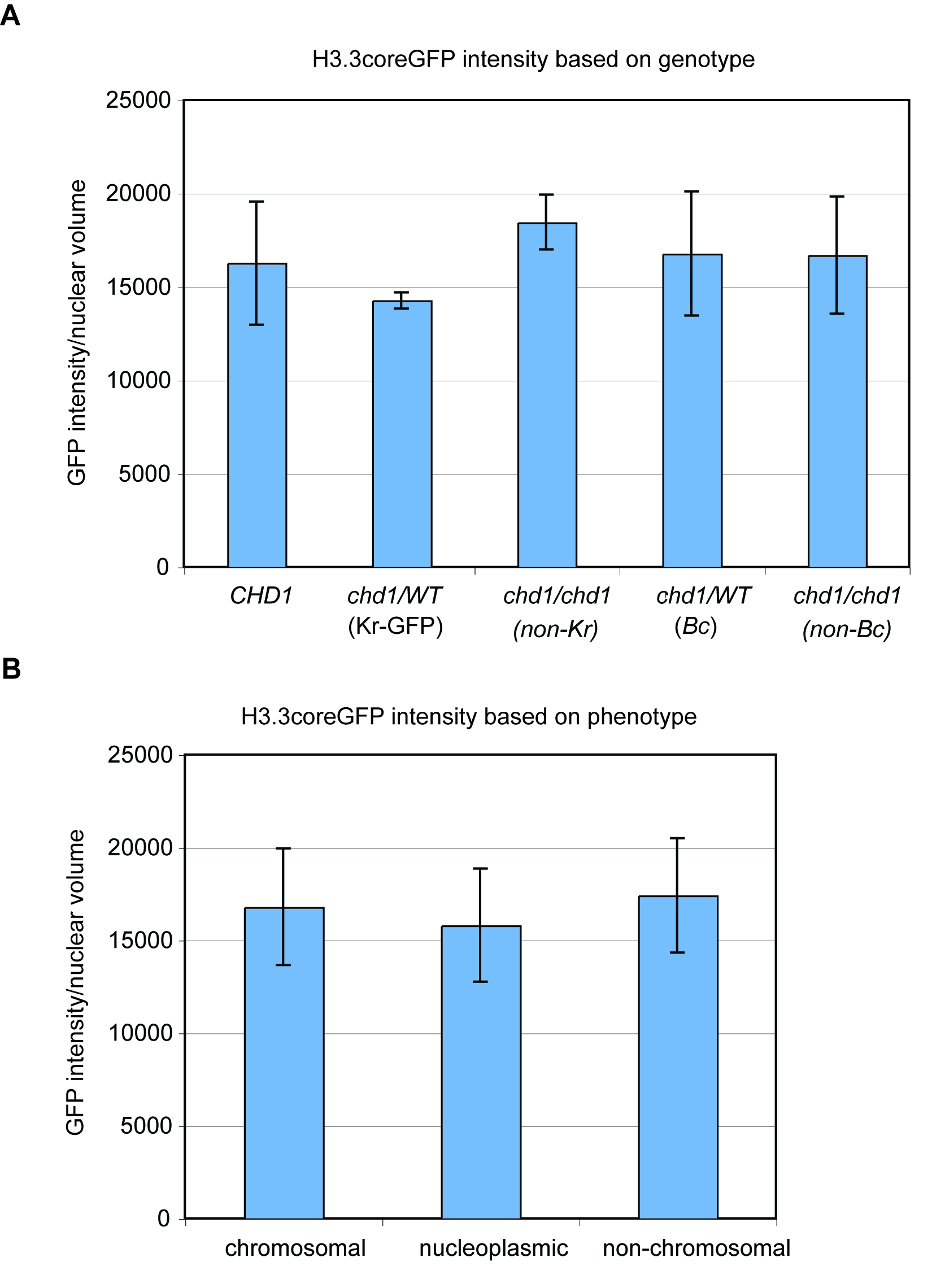

Supplement: Figure S1 — The chd15 mutation does not effect H3.3core-GFP expression. Total fluorescent intensity and volume of polytene nuclei were determined by analysis of confocal z-stacks using Volocity software. Average fluorescent intensity/nucleus (arbitrary units; +/− Std. Dev.) are shown for (A), the indicated genotypes and (B), the indicated phenotypes. In (A), non-Bc and non-Kr refer to the balancer chromosomes in the parental chd1 strains used to generate the homozygous nulls. (TIF) [file pgen.1002811.s001.tif]

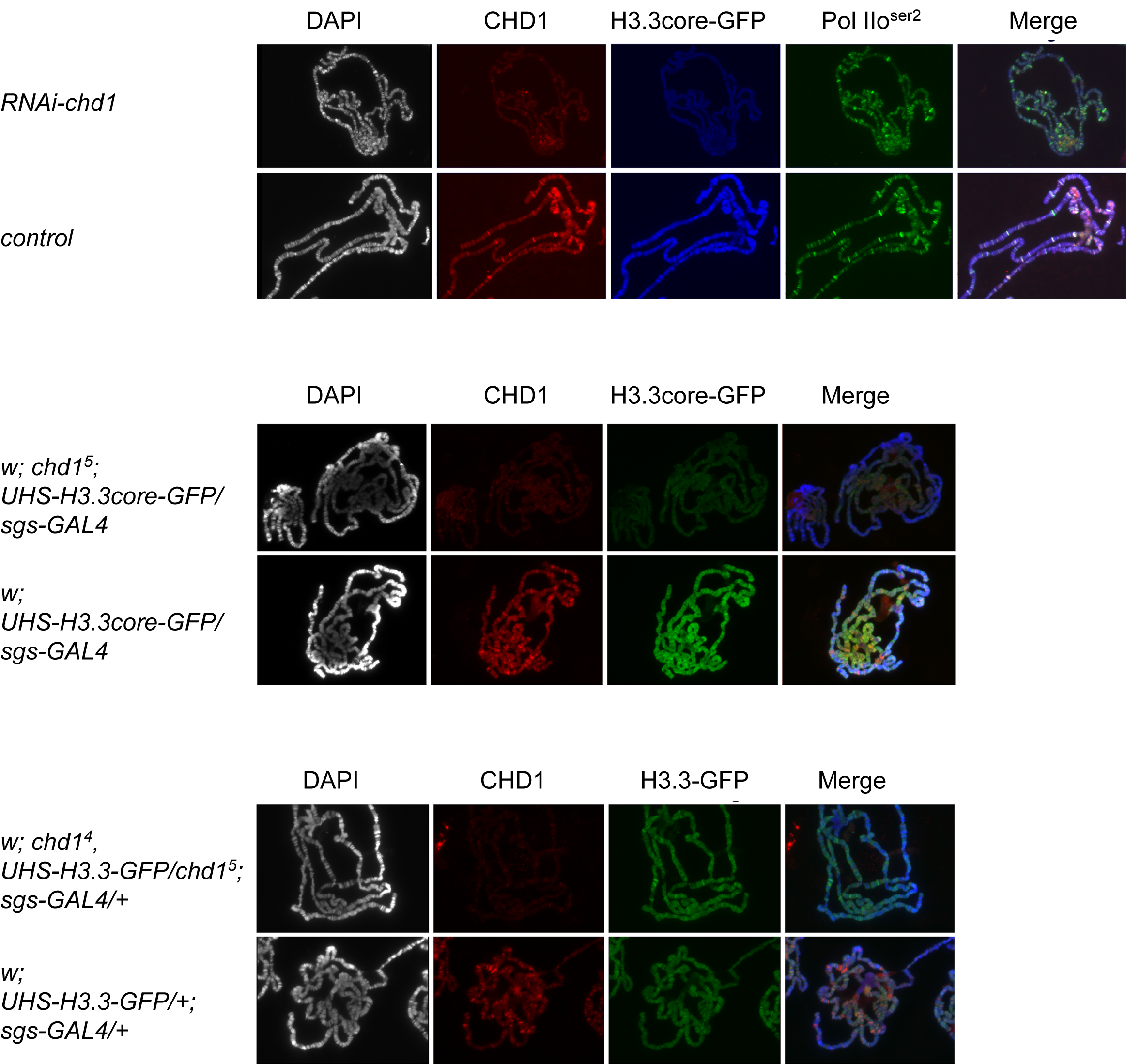

Supplement: Figure S2 — Loss of Chd1 affects localization of H3.3core-GFP but not H3.3-GFP. (A) Levels of H3.3coreGFP are reduced on polytene chromosomes of RNAi-chd1 expressing larvae (VDRC26277 driven by AB1-gal4; [70] ) as compared to those from control larvae. Chd1 (red), Pol IIoser2 (green), GFP (blue), DAPI (white in left panel, not included in merge). (B) Levels of H3.3coreGFP are reduced on polytene chromosomes derived from chd1 mutant larvae as compared to control larvae. (C) In contrast, levels of full length H3.3-GFP remain similar on polytenes derived from chd1 mutant larvae as compared to control larvae. Both chd15 and chd14 are null alleles [21]. Chd1 (red), GFP (green), DAPI (white in left panel and blue in merge). (TIF) [file pgen.1002811.s002.tif]

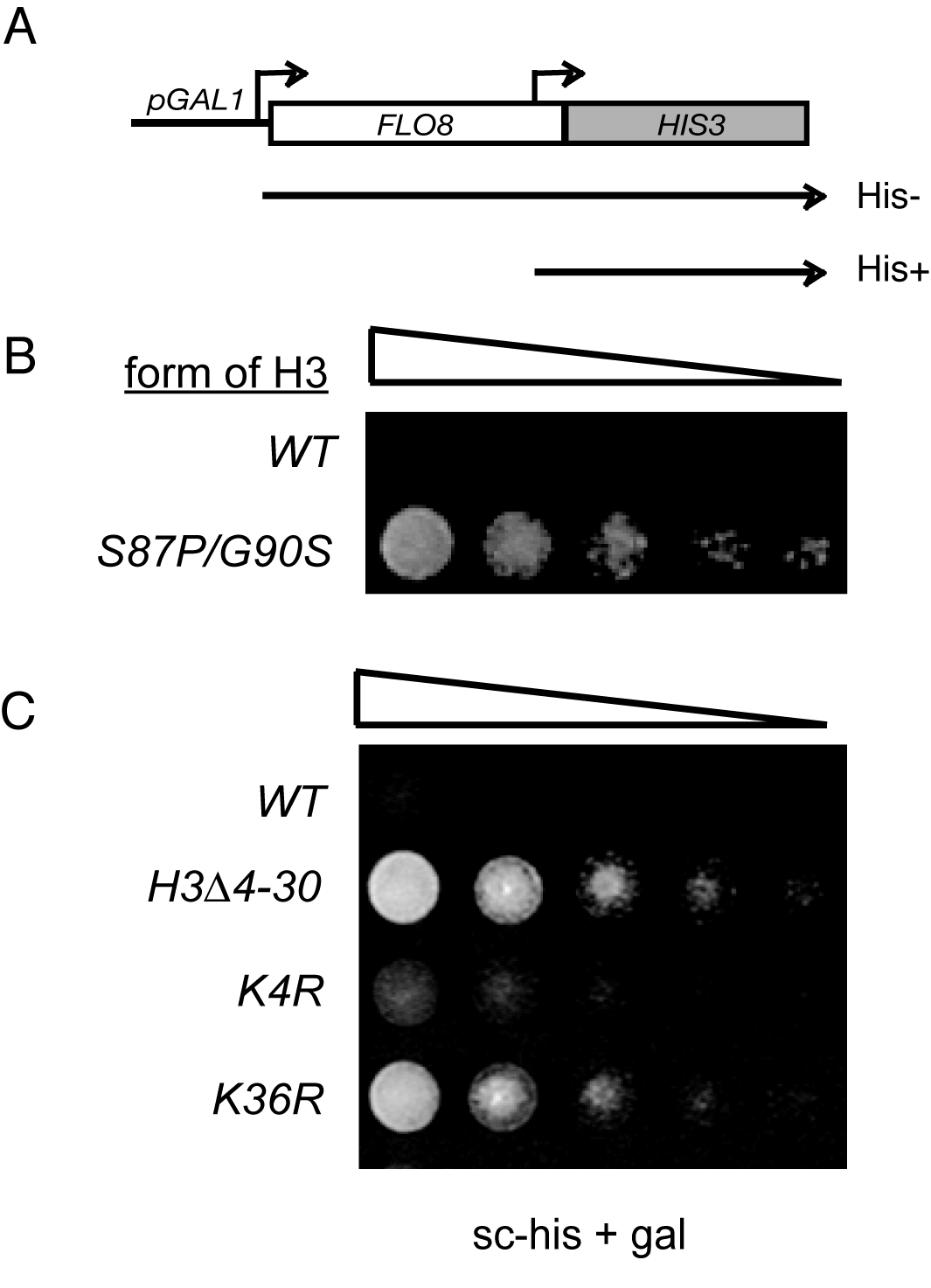

Supplement: Figure S3 — The histone H3-S87P/G90S mutation causes a cryptic initiation phenotype. (A) Diagram of the pGAL1-FLO8-HIS3 reporter gene. Transcription initiation from the normal FLO8 start site produces a transcript in which HIS3 in out of frame and not translated. Internal initiation from within FLO8 produces in frame transcripts and a His+ phenotype as indicated by growth on media lacking histidine. Strain GHY2010 was transformed with CEN LEU2 hht2 HHF2 plasmids carrying the indicated mutations and plated to 5FOA to select for cells that had lost the wild type CEN URA3 HHT2 HHF1 plasmid. These cells were subsequently grown in liquid culture, adjusted to 1×10^7^ cells/ml and 5-fold serial dilutions were spotted to SC-His+Gal media and incubated at 30°C for 3 days. (B) The H3-S87P/G90S mutation causes a cryptic initiation phenotype. (C) Positive and negative controls. As demonstrated previously [28], the H3Δ4-30 and H3K36R mutations cause a cryptic initiation phenotype whereas H3K4R mutation does not. (TIF) [file pgen.1002811.s003.tif]

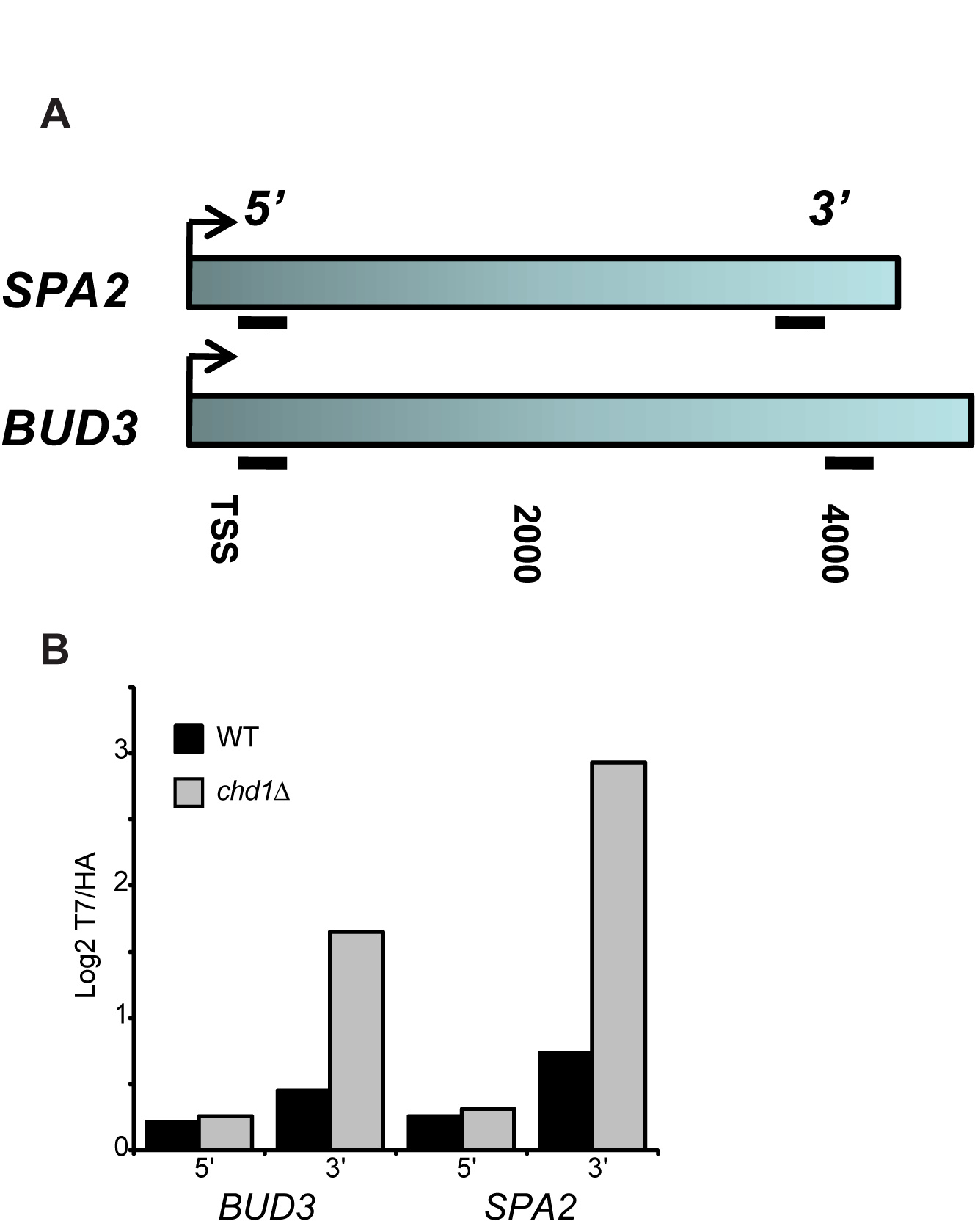

Supplement: Figure S4 — Chd1 affects H3 turnover in an independent turnover assay. (A) Schematic showing the locations of 5′ and 3′ q-PCR primers within the indicated genes. (B) H3 turnover was measured using the system described in [51]. Briefly, yeast carrying H3-HA were arrested by nutrient depletion, and Cre-Lox recombination was used to recombine out the HA tag to yield H3-T7 expression. Yeast were released into cell cycle arrest in benomyl-nocodazole, and H3-HA and H3-T7 were isolated by ChIP. T7/HA ratio (indicating H3 replacement) was calculated by q-PCR at the indicated locations for wild type and chd1Δ yeast, as indicated. As found using the pGAL-based turnover system, loss of Chd1 resulted in increased 3′ turnover over these long coding regions. (TIF) [file pgen.1002811.s004.tif]

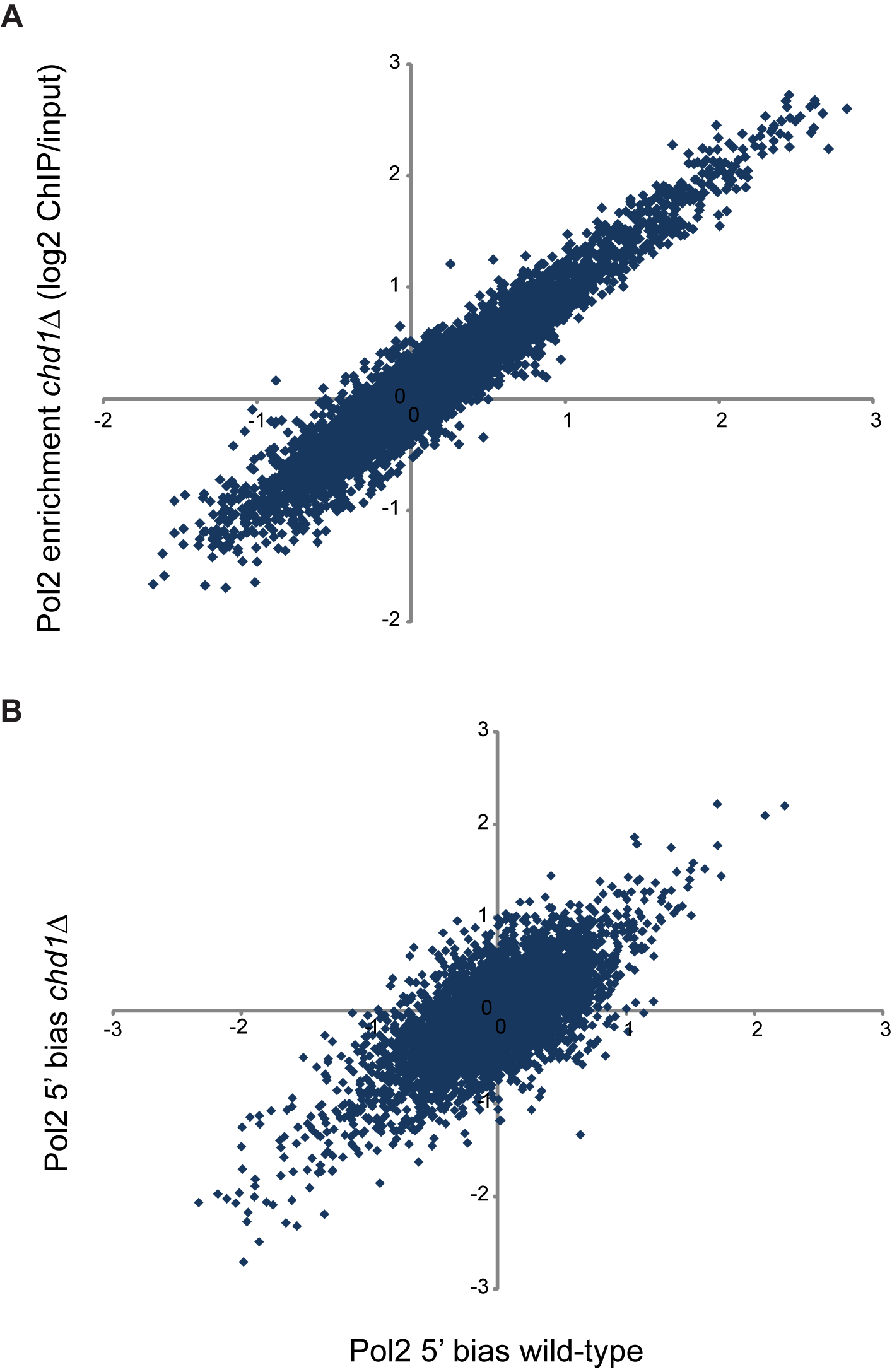

Supplement: Figure S5 — Chd1 has minimal effects on global transcription. (A) RNA Pol2 was mapped genome-wide in wild type and chd1Δ, and RNA Pol2 enrichment was averaged for all genes. Scatterplot shows average RNA Pol2 enrichment for all genes, comparing wild type (x axis) and chd1Δ (y axis). (B) 5′ bias in RNA Pol2 (defined as the 5′ RNA Pol2 enrichment over the 3′ RNA Pol2 enrichment) is scatterplotted for wild type and mutant as indicated. Note strong correlation with slope = 1, indicating no systematic bias in RNA Pol2 localization patterns in chd1Δ mutants. (TIF) [file pgen.1002811.s005.tif]

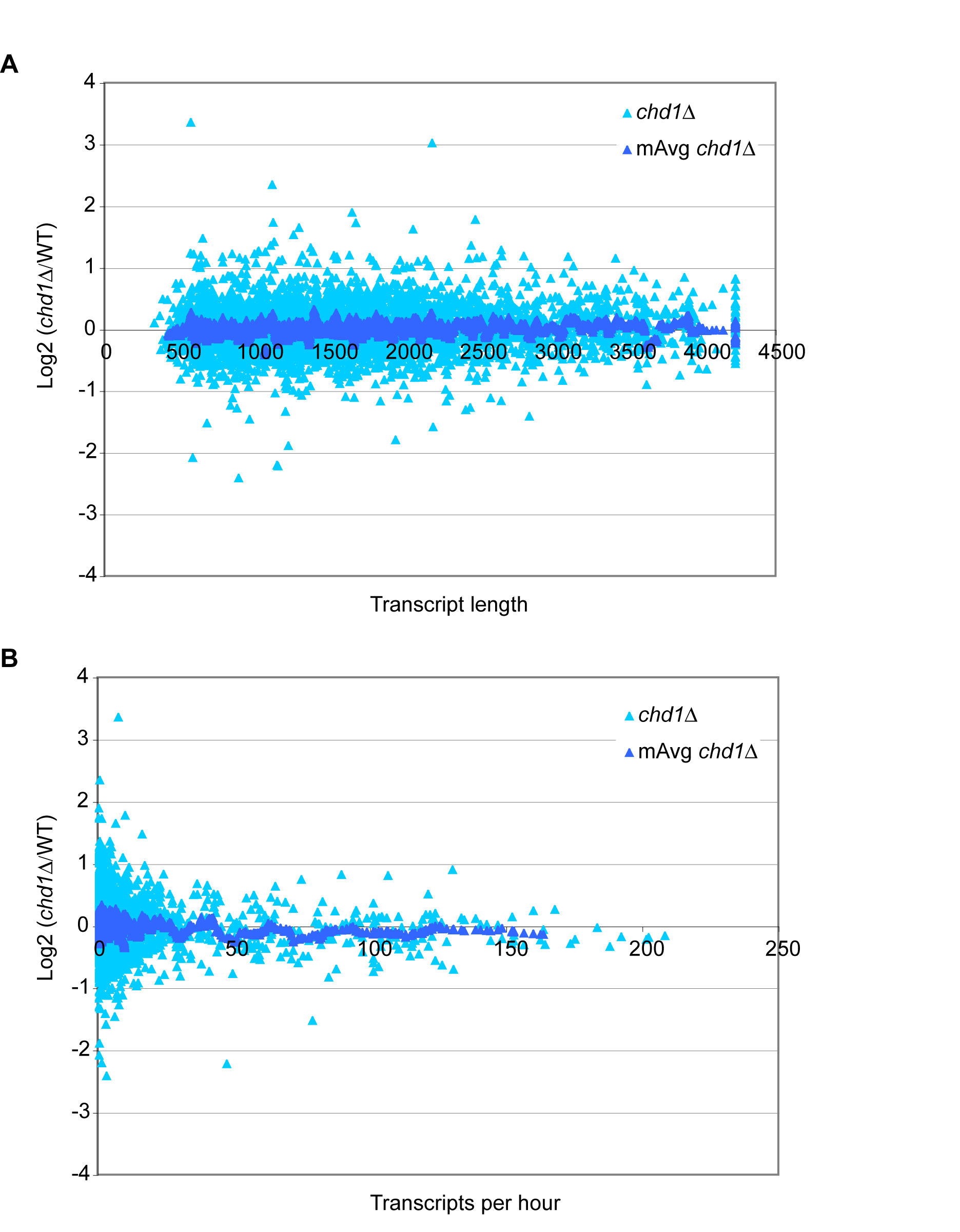

Supplement: Figure S6 — Lack of correlation between transcript length, transcription frequency and changes in mRNA expression in chd1 mutant. (A) Scatterplot comparing a 20 gene moving average of Log2 (mut/WT) chd1 expression microarray data to transcript length. (B) Scatterplot comparing a 20 gene moving average of Log2 (mut/WT) chd1 expression microarray data to gene transcription frequency in wild type cells. (TIF) [file pgen.1002811.s006.tif]

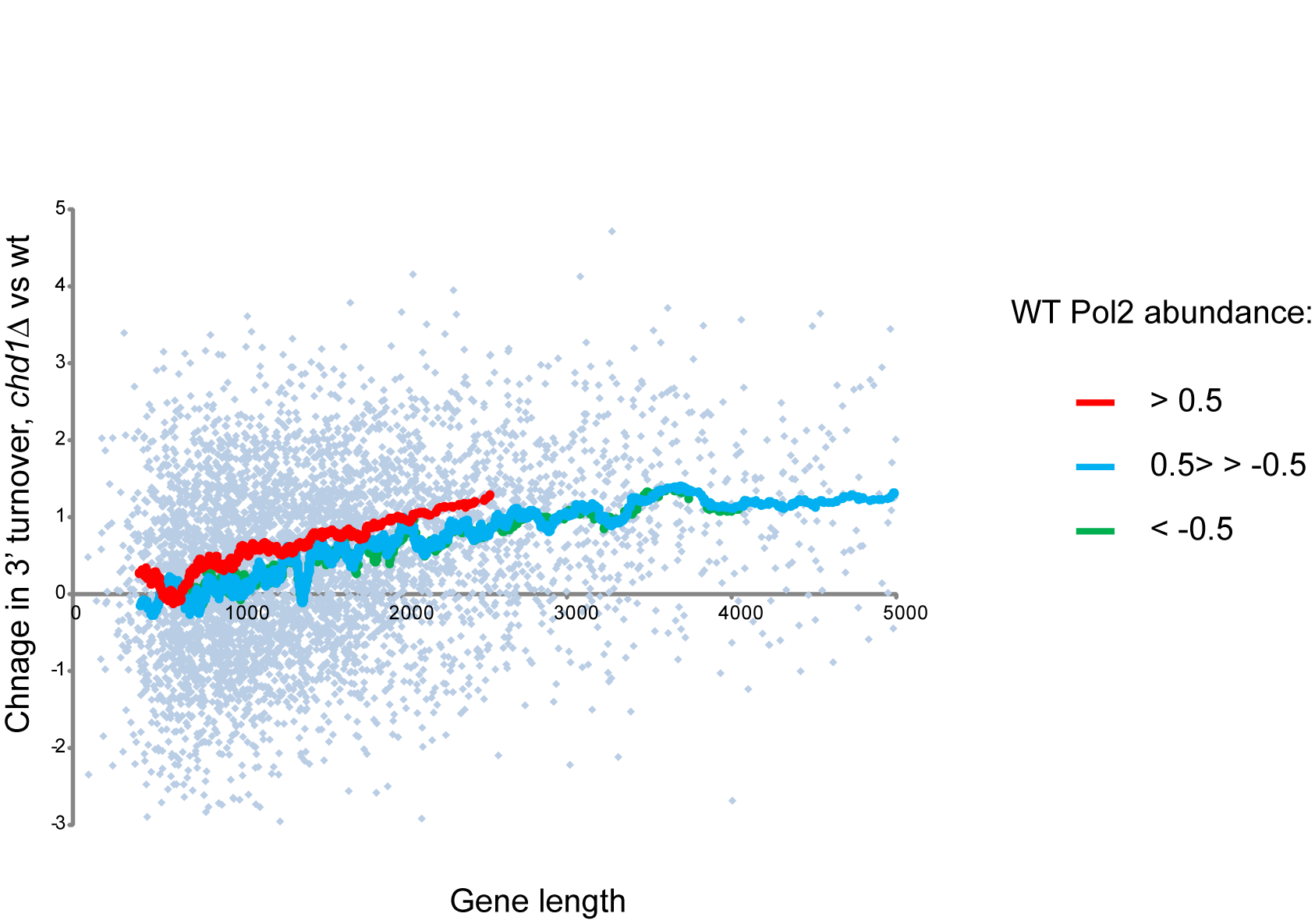

Supplement: Figure S7 — Chd1 effects on 3′ histone replacement are greater at highly transcribed genes. The change in histone replacement at the 3′-CDS (last 500 bp of coding regions) between wild type and chd1Δ yeast was calculated, and this value is scatterplotted against gene length. Within this scatterplot, we generated 80 gene running window averages for those genes transcribed at low (green), middle (blue), and high (red) levels in wild type cells based on genome-wide Pol2 ChIP-chip [71]. Note that the red line is consistently higher than the blue or green lines, indicating that after correcting for gene length, genes with higher transcription rates exhibit greater Chd1-dependent stabilization of 3′ nucleosomes. (TIF) [file pgen.1002811.s007.tif]

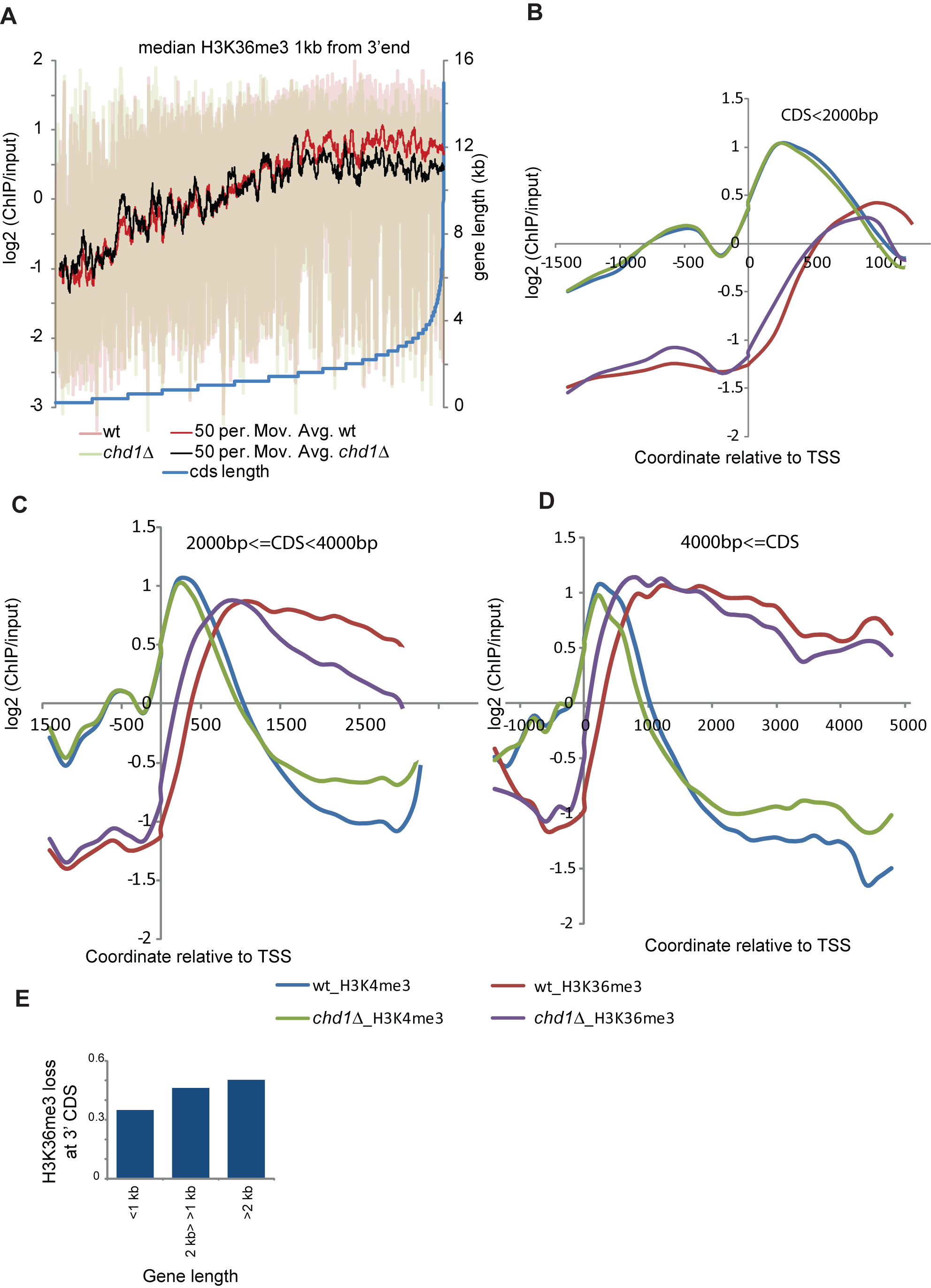

Supplement: Figure S8 — Histone modification changes correlate with gene length. (A) H3K36me3 levels at the 3′ 1 kb of genes is shown for wild type and mutants, as indicated. Lines show a 50 gene running window average. (B–D) Averaged H3K4me3 and H3K36me3 data for wild type and mutant yeast is shown for short (B), long (C), and extremely long (D) genes. (E) Average gain in H3K36me3 levels at the 3′ 500 bp of genes was averaged for the three indicated gene length classes. (TIF) [file pgen.1002811.s008.tif]
