# Supplementary material for: A Key Role for Chd1 in Histone H3 Dynamics at the 3′ Ends of Long Genes in Yeast
Source: PLoS Genet. 2012 Jul 12;8(7):e1002811. doi: 10.1371/journal.pgen.1002811 (PMC3395613; doi:10.1371/journal.pgen.1002811)
Supplement: Table S1 — Yeast strains used in the study. (DOC) [file pgen.1002811.s009.doc]

**Table S1.** Yeast strains used in this study

Strain Genotype Source

GHY 2315 *MAT***a** *bar1∆::natMX (hht1-hhf1)∆::HIS3* This study

*his3∆1 lys2∆0* *leu2∆0 ura3∆0*

GHY 2317 *MAT***a** *bar1∆::natMX (hht1-hhf1)∆::HIS3* This study

*his3∆1 lys2∆0 leu2∆0 ura3∆0 chd1∆::kanMX*

GHY279 *MATα* *his3∆200 lys2-128δ ura3-52 leu2∆1*  Quan and Hartzog,

*chd1∆::HIS3 trp1∆63* Genetics, 184:321-34

2010.

FY120 *MAT***a**  *his4-912δ lys2-128δ leu2∆1 ura3-52* Fred Winston

GHY823 *MAT***a** *his3∆200 leu2∆1 lys2-128**δ ura3-52 trp1∆63* This study

*(hht1-hhf1)∆::LEU2 (hht2-hhf2)**∆::HIS3*

[pDM9=*(HHT1-HHF1)URA3*]

GHY1806 *MAT***a** *his3∆200 leu2∆1 lys2-128δ ura3-52 trp1∆63* This study

*(hht1-hhf1)∆::LEU2 (hht2-hhf2)∆::HIS3 chd1∆::HIS3*

[pDM9=*(HHT1-HHF1)URA3*]

GHY2010 *MAT***a** *his3∆200 leu2∆1 lys2-128δ ura3-52 trp1∆63* Quan and Hartzog,

*(hht1-hhf1)∆::LEU2 (hht2-hhf2)∆::NAT* Genetics, 184:321-34

*pGAL1-FLO8-HIS3::KANMX* 2010.

[pDM9=*(HHT1-HHF1)URA3*]

GHY2523 *MAT***a** *bar1∆::natMX (hht1-hhf1)∆::HIS3* This study

*top1∆::kanMX his3∆1 lys2∆0 leu2∆0 ura3∆0*

GHY2525 *MAT***a** *bar1∆::natMX (hht1-hhf1)∆::HIS3* This study

*CHD1∆::kanMX top1∆::LEU2 his3∆1 lys2∆0*

*leu2∆0 ura3∆0*

NKI2215 *MAT***a** *his3**∆200 leu2∆0 trp1∆63 ura3∆0 met15∆0* Verzijlbergen, PLoS

(*hht1-hhf1)∆::MET15 bar1::HisG*  Gen 7(10):

*HIS3:P_TDH3_-CRE-EBD78* e1002284, 2011.

*hht2::HHT2-LoxP-HA-HPHMX-LoxP-T7*

NKI2216     *MAT***a** *his3∆200 leu2∆0 trp1∆63 ura3∆0 met15∆0*  Verzijlbergen, PLoS

(*hht1-hhf1)∆::MET15 bar1::HisG*  Gen 7(10):

*HIS3:P_TDH3_-CRE-EBD78*  e1002284, 2011.

*hht2::HHT2-LoxP-T7-HPHMX-LoxP-HA*

NKI2308     NKI2215 *chd1∆::KanMX*     This study

NKI2309   NKI2216 *chd1∆::KanMX*   This study
