# Supplementary material for: A Key Role for Chd1 in Histone H3 Dynamics at the 3′ Ends of Long Genes in Yeast
Source: PLoS Genet. 2012 Jul 12;8(7):e1002811. doi: 10.1371/journal.pgen.1002811 (PMC3395613; doi:10.1371/journal.pgen.1002811)
Supplement: Table S2 — Plasmids used in this study. (DOC) [file pgen.1002811.s010.doc]

**Table S2.** Plasmids used in this study

Plasmid Source

pGAL-H4-FlagH3 *HHF1-GAL10/1-Flag-HHT1 CEN URA3* *Amp^r^* This study

pJH18 *HHT2-HHF2 TRP1 CEN Amp^r^* Hsu *et al.*,

Cell 102:279- 291

pJH18-AO6 *hht2-S87P/G90S-HHF2 TRP1 CEN Amp^r^* This study

pJH18-Δ4-30, S87P/G90S

*hht2Δ4-30-S87P/G90S-HHF2 TRP1 CEN Amp^r^* This study

pRS314 *TPR1 CEN Amp^r^* Sikorski and Hieter Genetics 122:19-27

pRM430 *hht2Δ4-30 HHF2 TRP1 CEN* AmpR Michael Grunstein

MBB257 *hht2-K4R HHF2 TRP1 CEN* AmpR Mary Bryk

MBB286 *hht2-K36R HHF2 TRP1 CEN* AmpR Mary Bryk

pFXO4-K56G *hht2-K56G* *HHF2 TRP1 CEN* AmpR Michael Grunstein

pWZ414-F13-K79A Kevin Struhl

*hht2-K79A HHF2 TRP1 CEN* AmpR
