# Supplementary material for: A Key Role for Chd1 in Histone H3 Dynamics at the 3′ Ends of Long Genes in Yeast
Source: PLoS Genet. 2012 Jul 12;8(7):e1002811. doi: 10.1371/journal.pgen.1002811 (PMC3395613; doi:10.1371/journal.pgen.1002811)
Supplement: Table S3 — Oligonucleotides used for qPCR Analysis of Chromatin Immunoprecipitates. The indicated oligonucleotide pairs were used in initial analyses and verification of histone turnover ChIP experiments. (DOC) [file pgen.1002811.s011.doc]

**Table S3. Oligonucleotides used for qPCR Analysis of Chromatin Immunoprecipitates**

| Gene name | Length  (bp) | PCR product  position | Oligo | Sequence |
| --- | --- | --- | --- | --- |
|  |  |  |  |  |
| *GAL1* | 1587 | promoter | OGH 175 | GGTAATTAATCAGCGAAGCGATGATTT |
|  |  |  | OGH 172 | TGCGCTAGAATTGAACTCAGGTAC |
|  |  |  |  |  |
|  |  | ORF1 | OGH 216 | TAACAAGTTTGAAACCGCCC |
|  |  |  | OGH217 | TCGTGCTCGATCCTTCTTTT |
|  |  |  |  |  |
|  |  | ORF2 | OGH 154 | TGCCGACGAAGACTTTTTCA |
|  |  |  | OGH 155 | TCATATAGACAGCTGCCCAATG |
|  |  |  |  |  |
|  |  |  |  |  |
| *PMA1* | 2757 | promoter | OGH 738 | CGAGACCGTTTATCCATTGC |
|  |  |  | OGH 739 | TTCGTCACCGGTCATAATTG |
|  |  |  |  |  |
|  |  | ORF1 | OGH 346 | CGACGACGAAGACAGTGATAACG |
|  |  |  | OGH 347 | ATTGAATTGGACCGACGAAAAACATAAC |
|  |  |  |  |  |
|  |  | ORF2 | OGH 348 | AAGTCGTCCCAGGTGATATTTTGCA |
|  |  |  | OGH 349 | AACGAAAGTGTTGTCACCGGTAGC |
|  |  |  |  |  |
|  |  | ORF3 | OGH 352 | CTATTATTGATGCTTTGAAGACCTCCAG |
|  |  |  | OGH 353 | TGCCCAAAATAATAGACATACCCCATAA |
|  |  |  |  |  |
|  |  |  |  |  |
|  |  |  |  |  |
| *RPL26B* | 861 | ATG | L-87 | AAAGTATTAGCCCGTTCTCAGTGCTTC |
|  |  |  | L-88 | CGGAAATTCACTCATGTTTTCTATCGT |
|  |  |  |  |  |
|  |  | ORF | L-89 | GCTGACATAAATTCCGGCTTTAGAATC |
|  |  |  | L-90 | CAATGAGCACATAATTTTCATCCACAC |
|  |  |  |  |  |
|  |  |  |  |  |
| *PHO5* | 1404 | ATG | OGH 940 | TCGACATCGGCTAGTTTGCCTAA |
|  |  |  | OGH 941 | AGAGCAAGCAAATTCGAGATTACCA |
|  |  |  |  |  |
|  |  | ORF | OGH 945 | GATGCCAATGATGACATTGTAAATG |
|  |  |  | OGH 946 | TGCACACCACGAGAATAAAGTACTA |
|  |  |  |  |  |
|  |  |  |  |  |
| *Chr V* |  | ARS504 | OGH 232 | GGCTGTCAGAATATGGGGCCGTAGTA |
|  |  |  | OGH 233 | CACCCCGAAGCTGCTTTCACAATAC |
|  |  |  |  |  |
|  |  |  |  |  |
| *Tel 0.5* |  |  | LOU189 | GCGTAACAAAGCCATAATGCCTCC |
|  |  |  |  |  |
|  |  |  | LOU190 | CTCGTTAGGATCACGTTCGAATCC |
|  |  |  |  |  |
| *SNF6* | 999 | 5’ CDS | OGH1086 | GCGGAGGAAAACAACTATCAGTACG |
|  |  |  | OGH1087 | ATATTGCTGAGCTGTTCTGGTCTAAGT |
|  |  |  |  |  |
|  |  | 3’ CDS | OGH1088 | AAGTCCTCATGCAACTGCAAC |
|  |  |  | OGH1089 | ATACAGCATCAAGATCTCCAAATTC |
|  |  |  |  |  |
|  |  |  |  |  |
| *CAR1* | 1002 | 5’ CDS | OGH1079 | GTCTGCAAACAAGCATAGAGGATTT |
|  |  |  | OGH1080 | TTTAGCCTTGACACCGTCTATCATAA |
|  |  |  |  |  |
|  |  | 3’ CDS | OGH1081 | GTGTAGACCCATTATACATTCCTGCTA |
|  |  |  | OGH1082 | GAATAGCCAGATCAGGGTTACATTC |
|  |  |  |  |  |
|  |  |  |  |  |
| *RIM4* | 2142 | 5’ CDS | OGH1100 | CGACTCTGAGCTGGTTATCAGAGAG |
|  |  |  | OGH1099 | TGGATGTGGAAGTGGTAGTGACTGT |
|  |  |  |  |  |
|  |  | 3’ CDS | OGH1101 | CCATTGCATCCTTCTCAAGGTTCT |
|  |  |  | OGH1102 | GAACATGGGTACTGCCATGATTAGC |
|  |  |  |  |  |
|  |  |  |  |  |
| *UME6* | 2511 | 5’ CDS | OGH1093 | CTCTCTGCTTTCGATGGAAACAACC |
|  |  |  | OGH1094 | GAAATTGTAGGATGGCGGGAGTTAG |
|  |  |  |  |  |
|  |  | 3’ CDS | OGH1097 | TGCTACTTCCTCAACGTCTCAAGGT |
|  |  |  | OGH1096 | AAATCTGGTTTGAACGCGTCATAGT |
|  |  |  |  |  |
|  |  |  |  |  |
| *RMD11* | 3441 | 5’ CDS | OGH1113 | ATAATCCAACGAAGCATCAGCACCT |
|  |  |  | OGH1114 | CTTTTCGCTGACTCGTTTAGAGCAA |
|  |  |  |  |  |
|  |  | 3’ CDS | OGH1117 | GACAACAACAAAGATGTGGACGAAA |
|  |  |  | OGH1118 | CATGTTGCATTTCTGGATCATCGTA |
|  |  |  |  |  |
|  |  |  |  |  |
| *IKI3* | 4050 | 5’ CDS | OGH1107 | GTTGGCTTCATTCAACATTCAGACT |
|  |  |  | OGH1108 | ATTAAAGTTTCAGCTGGGTCCAAAC |
|  |  |  |  |  |
|  |  | 3’ CDS | OGH1111 | GTGCTCGTGGTAAGAAGGGAACTAT |
|  |  |  | OGH1112 | GTTTCGTCTACAAAGACCCTCAACA |
